# Corneal Toxicity of Mirvetuximab Soravtansine: Multimodal Imaging Features and Implications for Ophthalmologic Management

**DOI:** 10.3390/diagnostics16071107

**Published:** 2026-04-07

**Authors:** Francesco De Dominicis, Andrea Giudiceandrea, Martina Cocuzza, Simone Bruzio, Romina Fasciani, Luigi Mosca, Chiara Giudiceandrea, Matteo Salgarello, Epifanio Giudiceandrea, Filippo Amore, Stanislao Rizzo, Maria Vittoria Carbone, Vanda Salutari, Anna Fagotti, Tommaso Salgarello

**Affiliations:** 1 Eye Clinic, Fondazione Policlinico Universitario A. Gemelli IRCCS, Largo A. Gemelli 8, 00168 Rome, Italy; andrea.giudiceandrea@policlinicogemelli.it (A.G.); martina.cocuzza@guest.policlinicogemelli.it (M.C.); simone.bruzio@gmail.com (S.B.); romina.fasciani@policlinicogemelli.it (R.F.); luigi.mosca@policlinicogemelli.it (L.M.); stanislao.rizzo@policlinicogemelli.it (S.R.); tommaso.salgarello@policlinicogemelli.it (T.S.); 2 IAPB Italia ETS Foundation, Fondazione Policlinico Universitario A. Gemelli IRCCS, 00168 Rome, Italy; f.amore@iapb.it; 3 Università Cattolica del Sacro Cuore, 00168 Rome, Italy; chiara.giudiceandrea@unicatt.it (C.G.); matsalga16@gmail.com (M.S.); vanda.salutari@policlinicogemelli.it (V.S.); anna.fagotti@policlinicogemelli.it (A.F.); 4 San Paolo Hospital, University of Milan, 20122 Milan, Italy; 5 Woman, Child and Public Health Department, Fondazione Policlinico Universitario A. Gemelli IRCCS, 00168 Rome, Italy

**Keywords:** mirvetuximab soravtansine, corneal toxicity, ocular adverse events, antibody–drug conjugates

## Abstract

**Background:** Mirvetuximab soravtansine (MIRV) improves outcomes in FRα-positive, platinum-resistant ovarian cancer; however ocular adverse events (OAEs), particularly corneal epithelial toxicity, are frequent and warrant structured ophthalmologic monitoring. **Methods:** In this retrospective observational study, 31 consecutive patients receiving MIRV for FRα-positive gynecologic malignancies underwent standardized ophthalmic assessments at baseline and prior to each treatment cycle (every 21 days). The protocol included best corrected visual acuity (BCVA), slit-lamp biomicroscopy, anterior-segment optical coherence tomography (AS-OCT), corneal topography, and tear film analysis. OAEs were graded according to the Common Terminology Criteria for Adverse Events (CTCAE) v5.0, based on symptom severity and functional impairment. **Results:** OAEs occurred in all patients (31/31, 100%), predominantly grade 1–2. Corneal epithelial toxicity was documented in 28/31 patients (90.3%), while no grade ≥ 3 events were observed. Symptoms typically developed 7–14 days after the second infusion. AS-OCT and corneal topography consistently revealed epithelial microcysts and surface irregularities, which were usually detected during scheduled pre-cycle ophthalmologic evaluations. Tear-film instability (break-up time ≤ 5 s) developed in 19/31 patients (61.3%), generally within 10 days after the second infusion, and improved in all but 2 patients (6.5%) following prophylactic lubrication. Transient refractive changes occurred in 28/31 patients (90.3%) and were associated with a temporary BCVA reduction (mean nadir ~20/32 Snellen), followed by recovery during follow-up. **Conclusions:** MIRV-related ocular alterations are frequent but reversible and clinically manageable. Multimodal imaging combined with functional and refractive assessment provides sensitive markers of corneal epithelial toxicity and supports integrated ophthalmologic monitoring to preserve visual function and maintain oncologic treatment continuity.

## 1. Introduction

Epithelial ovarian cancer remains the leading cause of death from gynecologic malignancies worldwide, with more than 300,000 new cases and over 180,000 deaths each year [1]. Despite progress in cytoreductive surgery, platinum-based chemotherapy, and the introduction of PARP inhibitors, more than 70% of patients are still diagnosed at an advanced stage, and the majority eventually develop platinum-resistant disease [2]. In this setting, therapeutic options are limited, with modest response rates and a median overall survival rarely exceeding 12 months [3]. These data highlight the urgent need for innovative treatments capable of improving outcomes while maintaining an acceptable safety profile.

Antibody–drug conjugates (ADCs) have emerged as a novel therapeutic class that combines the target specificity of monoclonal antibodies with the potent cytotoxic activity of chemotherapeutic agents. Mirvetuximab soravtansine (MIRV) is an ADC designed to target folate receptor alpha (FRα), a cell-surface glycoprotein highly expressed in high-grade serous ovarian carcinomas, fallopian tube cancers, and primary peritoneal carcinomas, while largely absent in normal tissues [3,4]. The molecule consists of a humanized IgG1 monoclonal antibody conjugated via a cleavable disulfide linker to DM4, a maytansinoid tubulin inhibitor that disrupts microtubule dynamics and induces apoptosis following intracellular release [5,6]. Clinical trials, including the phase II SORAYA and phase III MIRASOL studies, demonstrated significant efficacy of MIRV in FRα-positive, platinum-resistant ovarian cancer and related gynecologic malignancies, leading to its regulatory approval in 2022 [7,8,9].

Alongside its clinical benefits, MIRV has been associated with a unique spectrum of ocular adverse events (OAEs). The most characteristic finding is microcystic epithelial keratopathy, often accompanied by blurred vision, photophobia, foreign body sensation, and dry eye, with an incidence of 40–50% in pivotal trials [8,9]. Although usually reversible and manageable with prophylactic eye care, these events may negatively affect quality of life and, in severe cases, threaten treatment continuity [10].

The pathogenesis of MIRV-related ocular effects remains incompletely understood. Current hypotheses point to an off-target effect of the DM4 payload, which may diffuse into the tear film and be taken up by corneal epithelial cells, altering their turnover and barrier function [11,12,13]. The reversibility and early onset of microcystic epithelial keratopathy support the hypothesis of a direct, dose-dependent, but transient epithelial disturbance rather than structural damage.

Despite increasing awareness, most published data on MIRV ocular safety derive from clinical trial reports with limited ophthalmologic detail. Systematic correlations between symptoms, imaging findings, and refractive changes remain limited in real-world clinical practice.

This study aimed to characterize the incidence, timing, clinical presentation, and multimodal imaging features of MIRV-related ocular toxicity in FRα-positive, platinum-resistant ovarian cancer. Special emphasis was placed on correlating clinical and functional data (best corrected visual acuity [BCVA], refractive parameters, and tear-film stability) with imaging findings from anterior segment optical coherence tomography (AS-OCT) and corneal topography, to identify early and reversible biomarkers of MIRV-related ocular toxicity and to support a structured multidisciplinary management approach.

## 2. Materials and Methods

### 2.1. Study Design and Setting

This retrospective observational study was conducted at the Department of Ophthalmology—Glaucoma Service, in collaboration with the Department of Gynecologic Oncology, Fondazione Policlinico Universitario A. Gemelli IRCCS—Università Cattolica del Sacro Cuore (Rome, Italy), and included patients treated with mirvetuximab soravtansine as part of routine clinical care.

Baseline and follow-up ophthalmologic data were retrospectively retrieved from clinical records of patients who initiated treatment starting from December 2024. All ophthalmologic assessments were performed according to routine clinical practice and were not introduced specifically for research purposes.

Systematic ophthalmologic monitoring was part of standard clinical management following national reimbursement approval of mirvetuximab soravtansine in April 2025. For the purposes of the present analysis, data collection was temporally censored in July 2025.

The ophthalmologic evaluation included baseline and follow-up visits comprising BCVA, slit-lamp biomicroscopy, AS-OCT, corneal topography, and tear film analysis, all performed as part of routine care.

**Figure 1:**
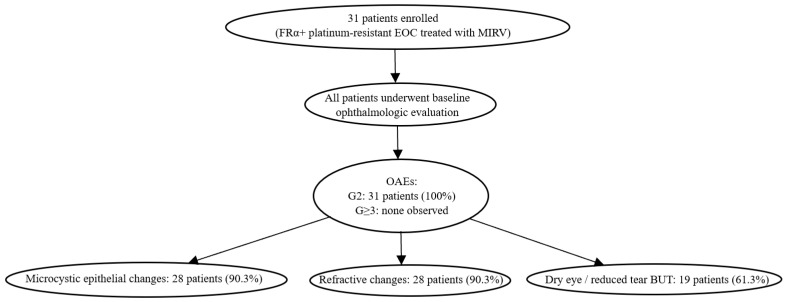
Study flowchart and ophthalmologic outcomes. Flow diagram illustrating patient inclusion, baseline ophthalmologic assessment, and OAEs during MIRV treatment (*n* = 31). All patients developed CTCAE grade 2 ocular adverse events. No grade ≥ 3 ocular toxicities were observed.

The overall study design and patient flow are summarized in Figure 1.

### 2.2. Patient Population

Eligible patients were women with histologically confirmed epithelial ovarian, fallopian tube, or primary peritoneal carcinoma, characterized by high FRα expression and platinum-resistant disease, who received MIRV as part of standard clinical care.

Patients with pre-existing severe ocular disease that could interfere with corneal assessment or visual function evaluation (e.g., keratoconjunctivitis sicca, corneal dystrophy, or recent ocular surgery within 3 months) were excluded.

A total of 31 consecutive patients were included in the present analysis. Demographic and clinical data were retrieved from electronic medical records for the retrospective component and prospectively collected for ongoing follow-up visits.

Baseline ophthalmologic examinations were largely unremarkable aside from age-appropriate findings. Mild ocular surface alterations, such as early dry eye signs or lens opacities, were occasionally observed; however, no clinically relevant corneal pathology was present prior to initiation of MIRV therapy.

All patients received MIRV according to standard oncologic indications. Given the advanced disease setting and ethical considerations related to treatment necessity, no untreated or non-prophylaxis control group was included.

### 2.3. Oncologic Treatment Protocol

All patients received MIRV intravenously at the standard dose of 6 mg/kg adjusted ideal body weight every 3 weeks, according to product labeling and institutional guidelines.

Dose modifications and treatment delays were applied in accordance with product labeling and institutional protocols, based on hematologic, hepatic, and ocular safety parameters. In particular, treatment adjustments related to ocular adverse events were guided by clinical symptoms and functional impairment, in line with oncologic recommendations, rather than by imaging findings alone.

Concomitant medications included corticosteroid premedication and antiemetic prophylaxis, administered according to standard oncologic protocols.

### 2.4. Ophthalmologic Assessment

A standardized ophthalmologic assessment protocol was applied at baseline (within 7 days before initiation of mirvetuximab soravtansine) and prior to each subsequent infusion, scheduled every 21 days in accordance with the oncologic treatment plan.

The same ophthalmologic protocol, visit timing, imaging acquisition settings, and assessment criteria were applied consistently across both the retrospective and prospective components of the study. All examinations were performed by the same dedicated ophthalmology team to ensure methodological consistency and minimize inter-observer variability.

All examinations were performed by the same dedicated ophthalmology team using consistent protocols, imaging acquisition settings, and assessment criteria, in order to ensure methodological consistency and minimize inter-observer variability.

The ophthalmologic evaluation integrated functional assessment with multimodal imaging to detect both symptomatic and subclinical ocular changes. Imaging findings were used for descriptive and monitoring purposes and did not independently determine toxicity grading or oncologic treatment decisions.

BCVA was measured using Snellen charts under standardized illumination conditions and converted to decimal notation for statistical analysis.

Slit-lamp biomicroscopy was performed using a Haag-Streit slit lamp (10–25× magnification; Haag-Streit AG, Koeniz, Switzerland), with diffuse and focal illumination. Fluorescein staining was added when clinically indicated to assess corneal epithelial integrity and tear film stability.

AS-OCT and corneal topography were acquired using a combined Placido–Scheimpflug device (MS-39, CSO, Florence, Italy; Phoenix software, version 4.1.60). AS-OCT was used to obtain high-resolution cross-sectional images and epithelial thickness maps, enabling detection of microcystic epithelial alterations. Corneal topography, integrating Placido-disk and Scheimpflug technologies, was used to evaluate corneal curvature, surface regularity, and induced astigmatic changes.

Imaging analysis was primarily qualitative, as validated quantitative thresholds for MIRV-related corneal toxicity are currently lacking.

Tear film assessment included non-invasive break-up time (NIBUT) and tear meniscus height measurements. In selected cases when clinically indicated, fluorescein break-up time and Schirmer I test were additionally performed. Refraction was conducted under non-cycloplegic conditions, reflecting routine clinical practice, with tear film status assessed prior to measurement to minimize variability related to ocular surface instability.

Anterior segment photography using digital slit-lamp imaging was performed for documentation and longitudinal follow-up of corneal epithelial alterations.

This comprehensive multimodal approach allowed correlation between subjective symptoms, functional impairment, and objective findings, enabling longitudinal monitoring of ocular toxicity throughout MIRV treatment.

### 2.5. Ophthalmologic Management Protocol

All patients received standardized prophylactic ocular therapy starting from the first mirvetuximab soravtansine infusion, consisting of preservative-free artificial tears administered at least three times daily. In cases of significant ocular surface dryness, nighttime lubricating gels or ointments were additionally prescribed.

Low-dose topical corticosteroids (fluorometholone 0.1%) were introduced in patients with clinically relevant symptoms or functional impairment, particularly when blurred vision, photophobia, BCVA reduction, or clinically significant corneal epithelial changes were observed on slit-lamp examination and/or anterior segment OCT. Overall, 28 out of 31 patients (90.3%) required topical steroid therapy during treatment. Corticosteroids were prescribed for short courses and tapered according to clinical response.

Protective eyewear with light-filtering lenses was recommended in patients reporting photophobia. Adjunctive topical agents, including trehalose-based antioxidant eye drops and vitamin A ointment, were added in selected cases with persistent epithelial alterations or tear film instability, based on clinical judgment.

Although management was tailored to individual patient presentation, the choice and escalation of adjunctive therapies were guided by clinical severity and patient-reported symptoms, following a consistent stepwise approach. Imaging findings were used to support clinical decision-making and longitudinal monitoring but did not independently dictate therapeutic escalation. This strategy allowed effective control of ocular adverse events in all cases, without requiring permanent discontinuation of mirvetuximab soravtansine.

### 2.6. Ethical Consideration

The study was conducted in accordance with the Declaration of Helsinki, and approved by Comitato Etico Territoriale Lazio Area 3 (CET Lazio Area 3), Fondazione Policlinico Universitario A. Gemelli IRCCS, Rome, Italy (approval number: Center ID 26989-1 (Protocol code: TOFA; RSO code: 4112), approval date: 12 February 2026). According to the Ethics Committee approval and in compliance with Italian regulations (Legislative Decree 196/2003, art. 110-bis) and GDPR provisions, informed consent was not required. A Data Protection Impact Assessment (DPIA) was also performed. All data and images included in the manuscript are fully anonymized and do not allow patient identification.

### 2.7. Outcome Measures

The primary outcome was the incidence of corneal epithelial toxicity detected by AS-OCT, including the presence of microcystic epithelial alterations.

Secondary outcomes included:

(i) The incidence and timing of OAEs, graded according to the Common Terminology Criteria for Adverse Events (CTCAE) v5.0, primarily based on symptom severity and functional impairment;
(ii) Changes in BCVA and refractive parameters during treatment;
(iii) The correlation between subjective ocular symptoms and instrumental findings obtained by AS-OCT, corneal topography, and tear film analysis;
(iv) The need for and response to ophthalmologic management strategies, including topical therapies, and their role in preventing treatment interruption or permanent discontinuation.

### 2.8. Statistical Analysis

Given the observational and primarily descriptive nature of the study, no formal sample size calculation was performed. All consecutive eligible patients evaluated during the study period were included in the analysis.

Continuous variables were expressed as mean ± standard deviation (SD) or median and interquartile range (IQR), as appropriate, based on data distribution. Categorical variables were summarized as absolute frequencies and percentages.

Changes in BCVA, refractive parameters, and tear-film metrics across visits were analyzed using paired statistical tests (paired *t*-test or Wilcoxon signed-rank test), depending on normality assumptions.

The timing of ocular adverse event onset was described descriptively in relation to treatment cycles. Associations between subjective ocular symptoms and instrumental findings were explored descriptively, given the limited sample size and the exploratory aim of the study.

All statistical tests were two-sided, and statistical significance was set at *p* < 0.05. Statistical analyses were performed using SPSS software (version 29.0; IBM Corp., Armonk, NY, USA).

## 3. Results

### 3.1. Patient Characteristics

Thirty-one women with FRα-positive, platinum-resistant epithelial ovarian, fallopian tube, or primary peritoneal carcinoma treated with MIRV were included in the analysis. The mean age of the cohort was 60.3 ± 8.4 years (range 35–75).

The majority of patients had high-grade serous carcinoma, consistent with the typical histologic profile of FRα-positive gynecologic malignancies. All patients had received at least one prior line of platinum-based chemotherapy and experienced disease progression within six months from the last platinum administration, fulfilling criteria for platinum-resistant disease [8,9].

Baseline ophthalmologic examinations were largely unremarkable, aside from age-appropriate findings. Mild ocular surface alterations, such as early dry eye signs or lens opacities, were occasionally observed; however, no clinically relevant corneal pathology or visual impairment was present prior to initiation of MIRV therapy.

### 3.2. Incidence and Timing of OAEs

Ocular adverse events were documented in all patients (31/31, 100%). Each patient experienced at least one grade 2 (G2) OAE according to CTCAE v5.0 criteria. Grade assignment was primarily based on symptom severity and functional impairment, including patient-reported visual symptoms and/or a reduction in BCVA to 20/40 or a loss of up to three Snellen lines from baseline in at least one eye. Instrumental findings were used to support clinical assessment but did not independently drive toxicity grading.

Corneal toxicity, specifically microcystic epithelial keratopathy, was detected in 28 out of 31 patients (90.3%), representing the most frequent ocular alteration associated with MIRV treatment.

This incidence appears higher than that reported in pivotal trials such as SORAYA and MIRASOL (Table 1), where rates of corneal adverse events ranged between approximately 40% and 50% [8,10]. However, this discrepancy should be interpreted with caution, as it likely reflects differences in ophthalmologic surveillance rather than increased intrinsic toxicity.

In the present real-world cohort, systematic and protocol-driven ophthalmologic monitoring, including multimodal imaging at predefined intervals, enabled the detection of subclinical corneal alterations that may have been underreported in trials relying primarily on symptom-driven assessments or less frequent specialist examinations.

Ocular symptoms typically developed within 7–14 days after the second MIRV infusion, in line with previously reported temporal patterns. Microcystic epithelial keratopathy was most commonly identified at the pre–third infusion visit, reflecting the timing of scheduled ophthalmologic evaluations rather than the precise onset of epithelial changes. The most frequently reported symptoms were blurred vision, photophobia, foreign body sensation, and ocular dryness.

### 3.3. Instrumental Findings

**Figure 2:**
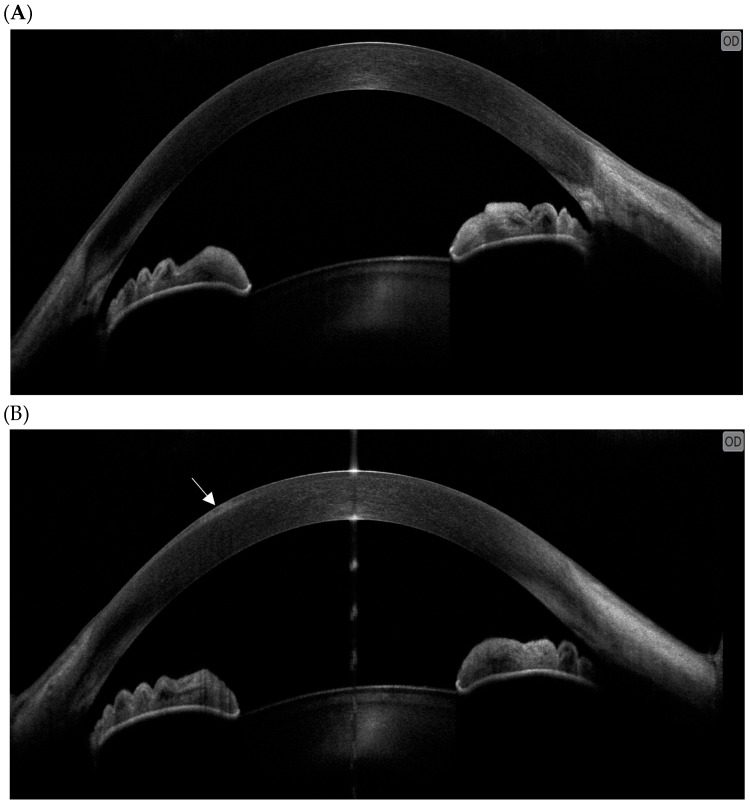
AS-OCT findings before and after MIRV treatment. (**A**) Baseline examination showing a regular, continuous corneal epithelium with preserved epithelial architecture. (**B**) After the second infusion of MIRV, AS-OCT reveals multiple intraepithelial hyperreflective microcystic changes associated with focal epithelial thickening and surface irregularity (white arrow), consistent with early drug-related corneal toxicity. Abbreviation: OD, right eye.

Anterior segment OCT demonstrated epithelial microcystic alterations in all cases classified as G2 OAEs (Figure 2A,B). Microcysts were predominantly paracentral in distribution, with occasional central extension in more advanced cases. Due to the absence of validated quantitative thresholds for MIRV-related epithelial toxicity, AS-OCT findings were analyzed qualitatively and used for longitudinal monitoring rather than for formal grading purposes.

**Figure 3:**
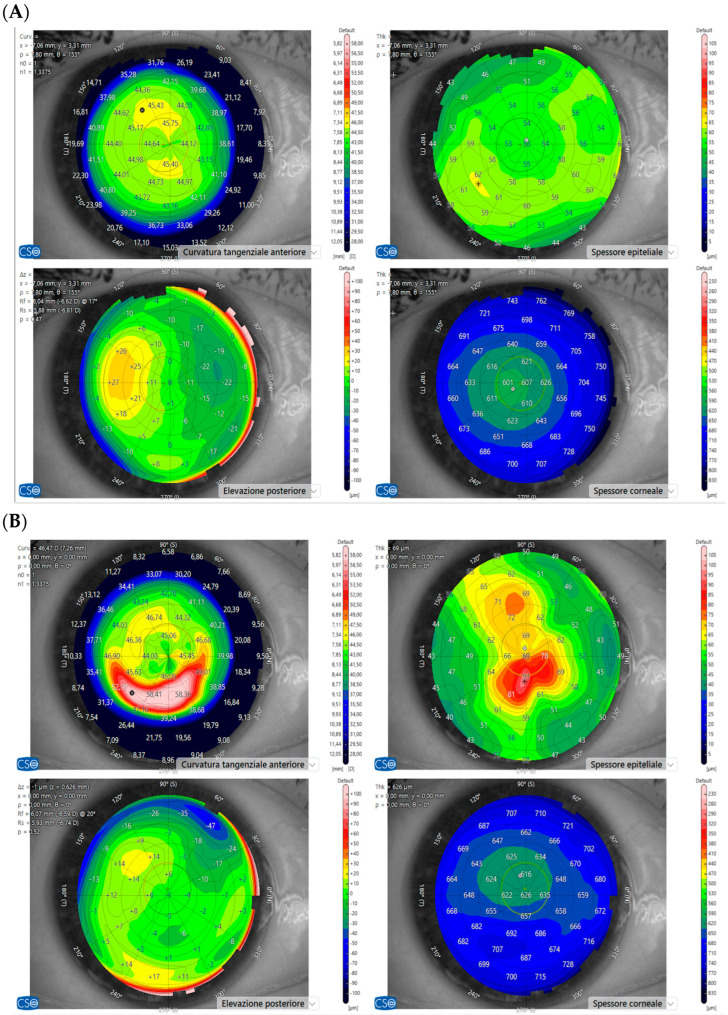
Corneal topography before and after MIRV treatment. (**A**) Baseline examination showing a regular and symmetrical anterior corneal curvature pattern and homogeneous epithelial thickness distribution. (**B**) After the second infusion of MIRV, corneal topography demonstrates central and paracentral irregular curvature patterns associated with localized epithelial thickness abnormalities, consistent with treatment-related epithelial surface instability. Symbols (* and ×) are part of the original device output and do not carry specific clinical meaning in this context.

Corneal topography revealed surface irregularities consistent with epithelial disorganization rather than stable refractive astigmatism (Figure 3A,B). These changes were characterized by variability across visits and lacked the regular, symmetric patterns typically associated with structural corneal astigmatism.

**Figure 4:**
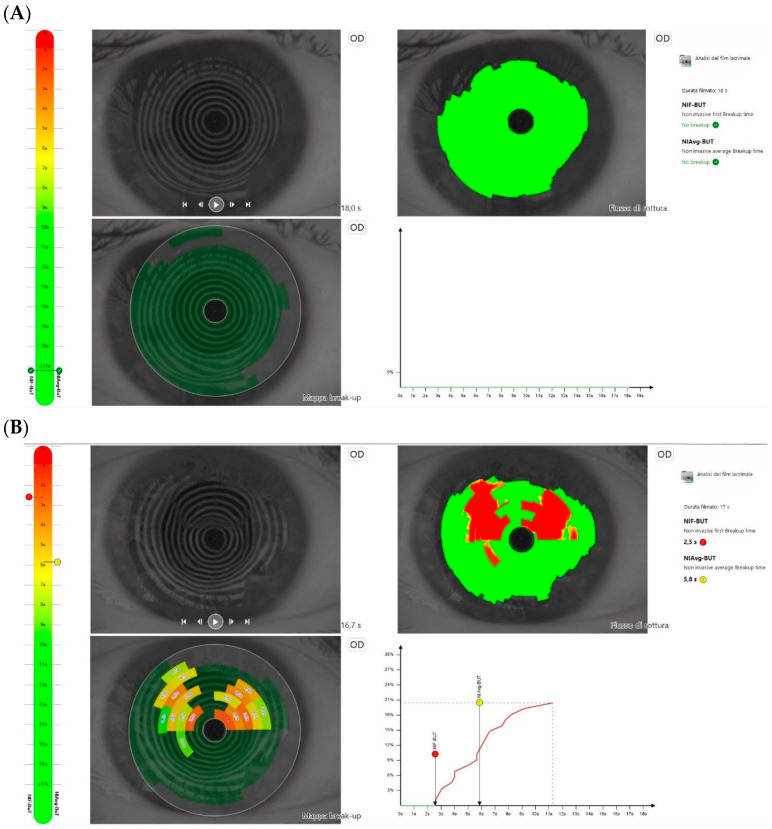
Tear film analysis before and after the second infusion of MIRV. (**A**) Baseline evaluation showing normal NIBUT and tear meniscus height. (**B**) Post-second infusion evaluation showing reduced NIBUT, indicative of tear film instability. Abbreviation: OD, right eye.

Tear-film analysis showed a reduced non-invasive break-up time (≤5 s) in 19 out of 31 patients (61.3%), typically emerging approximately 7–14 days after the second MIRV infusion (Figure 4A,B). Tear film instability often preceded or accompanied the detection of epithelial microcystic changes on AS-OCT, suggesting that ocular surface alteration represents an early manifestation of drug-related toxicity. Although prior chemotherapy exposure may have contributed to baseline ocular surface vulnerability, prophylactic initiation of preservative-free artificial tears resulted in marked improvement, with persistent tear instability observed in only 2 of 31 patients (6.5%) at subsequent follow-up visits.

**Figure 5:**
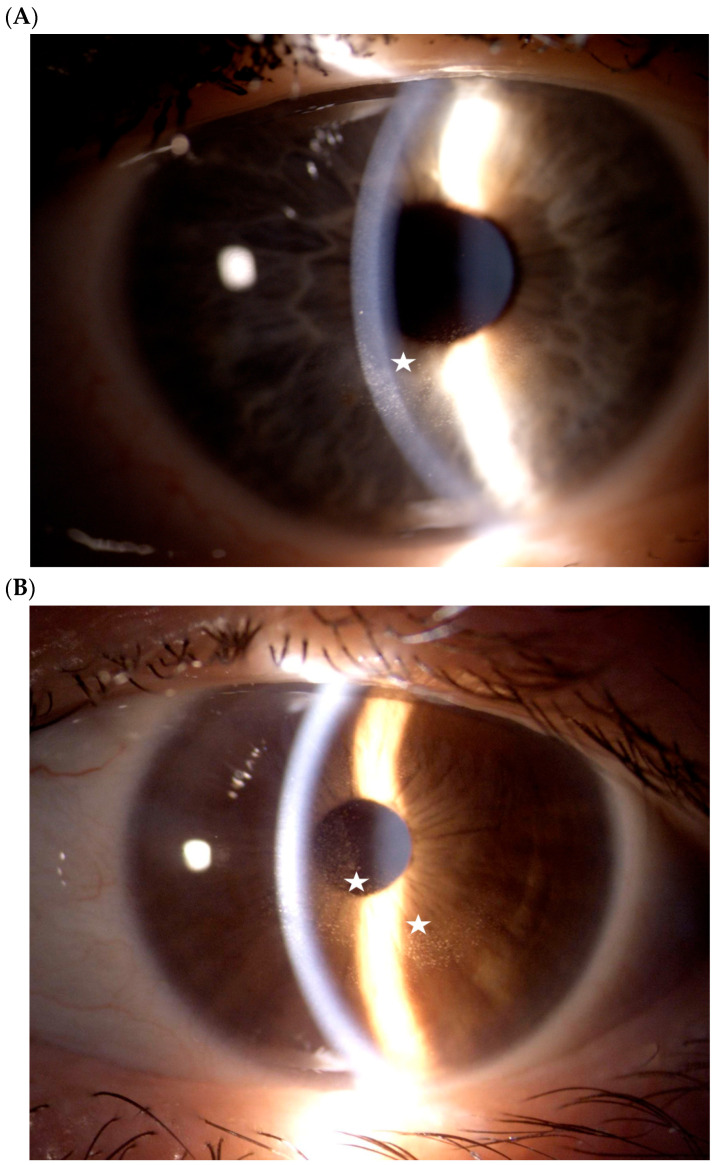
Anterior segment slit-lamp photographs in two representative patients with MIRV-related corneal epithelial alterations. (**A**) Paracentral epithelial microcystic changes with mild corneal haze (white star), with preserved stromal transparency. (**B**) Central epithelial microcystic changes associated with focal surface irregularity and reduced corneal clarity (white stars).

Anterior segment photography provided direct clinical documentation of microcystic epithelial keratopathy, illustrating the dynamic evolution of epithelial microcysts and associated changes in corneal transparency over the course of treatment (Figure 5A,B).

Refractive assessment further confirmed the presence of transient functional alterations. Among patients with epithelial keratopathy, 28 out of 31 (90.3%) developed new-onset or worsening irregular astigmatism, with a mean cylindrical shift of +1.50 ± 0.50 diopters.

These refractive changes were characterized by variability in the astigmatic axis across visits, consistent with an irregular and unstable corneal surface. Smaller spherical equivalent variations (ΔSE ±0.50–0.75 D) were also observed, generally hyperopic in cases with predominantly paracentral involvement and myopic when epithelial changes extended centrally.

Refraction was performed under non-cycloplegic conditions, reflecting routine clinical practice. Tear film status was assessed prior to refraction to minimize variability related to ocular surface instability. The observed refractive changes correlated with a temporary reduction in BCVA, with the worst recorded values reaching approximately 20/40 Snellen. Importantly, no patient developed permanent refractive abnormalities, and visual acuity recovered following resolution of epithelial alterations and optimization of ocular surface therapy. These findings support the interpretation that MIRV-related refractive changes are predominantly irregular and reversible, driven by epithelial disruption and tear film instability rather than by permanent corneal remodeling.

### 3.4. Management and Outcomes

All patients who developed G2 OAEs were managed according to the predefined ophthalmologic approach. Preservative-free artificial tears containing trehalose were prescribed in all cases as first-line therapy.

Overall, topical corticosteroid therapy (fluorometholone 0.1%) was required in 28 out of 31 patients (90.3%), administered as short courses and tapered according to clinical response. Adjunctive topical therapies were used in selected cases, including vitamin A ointment in 25.8% of patients. Protective eyewear with light-filtering lenses was recommended in patients reporting photophobia.

Importantly, no patient developed grade ≥ 3 toxicity, and no permanent discontinuation of mirvetuximab soravtansine was required due to OAEs.

At the last ophthalmologic follow-up available for the present analysis, 27 out of 31 patients (87.1%) showed functional recovery of BCVA, accompanied by marked improvement or resolution of microcystic epithelial keratopathy and associated symptoms. A minority of patients exhibited mild, residual epithelial irregularities; however, these findings remained clinically stable over time and did not result in further visual deterioration or impairment of quality of life.

Overall, systematic ophthalmologic monitoring combined with early, tailored management allowed effective control of MIRV-related ocular toxicity, enabling continuation of oncologic treatment without compromising safety or visual function.

### 3.5. Visual Function and Reversibility of Ocular Toxicity

Visual function impairment associated with MIRV treatment was transient and closely related to epithelial and tear film alterations rather than permanent structural damage. Reductions in BCVA were observed concomitantly with the development of microcystic epithelial keratopathy and tear film instability, typically after the second treatment cycle.

The mean worst BCVA recorded during treatment corresponded to a temporary decrease to approximately 20/32 Snellen, consistent with irregular astigmatism and epithelial surface disruption (Table 2). These functional changes were reversible and improved progressively following initiation of ophthalmologic management, particularly lubrication therapy and, when indicated, short courses of topical corticosteroids.

Refractive variations, predominantly characterized by induced irregular astigmatism and minor spherical equivalent shifts, were likewise transient. No patient developed permanent refractive error changes or required long-term optical correction attributable to MIRV-related toxicity.

At follow-up, the majority of patients demonstrated recovery of baseline visual acuity, paralleling the resolution or marked improvement of epithelial microcysts and stabilization of the tear film. Residual epithelial irregularities, when present, were mild, non-progressive, and not associated with further visual decline.

These findings support the concept that MIRV-related ocular toxicity primarily reflects a reversible, functional disturbance of the corneal epithelium and ocular surface. With systematic monitoring and timely intervention, visual impairment can be effectively controlled, allowing safe continuation of oncologic therapy without long-term visual sequelae.

## 4. Discussion

In this retrospective observational study of patients with FRα-positive, platinum-resistant gynecologic malignancies treated with mirvetuximab soravtansine, we observed a high incidence of OAEs, with microcystic epithelial keratopathy representing the most frequent and characteristic finding. The incidence of epithelial keratopathy in our cohort (90.3%) was substantially higher than that reported in pivotal trials such as SORAYA and MIRASOL (approximately 40–50%) [8,9,10]. This discrepancy should be interpreted cautiously and is likely attributable to differences in study design and monitoring intensity rather than to increased intrinsic ocular toxicity of MIRV.

In pivotal trials, ocular adverse events were primarily identified based on patient-reported symptoms and less frequent specialist evaluations. In contrast, our study implemented a systematic ophthalmologic monitoring protocol with scheduled multimodal imaging assessments, enabling detection of subclinical epithelial alterations that might otherwise remain unrecognized. Therefore, the higher incidence observed in our cohort most plausibly reflects detection bias related to proactive surveillance, rather than a true increase in drug-related toxicity.

Symptoms typically developed within 7–14 days after the second infusion, in agreement with previously reported temporal patterns [8,9,10], supporting the role of cumulative exposure to the DM4 payload in the pathogenesis of ocular surface changes. Importantly, no grade ≥ 3 OAEs were observed, and no patient required permanent treatment discontinuation due to ocular toxicity. These findings confirm that MIRV-related ocular toxicity is generally manageable in real-world clinical practice when promptly identified and appropriately treated [8,10,11].

From an instrumental standpoint, AS-OCT and corneal topography consistently documented epithelial microcysts and surface irregularities, while tear film instability was observed in 61.3% of patients, typically emerging within 10 days of the second infusion. Together, these findings highlight the sensitivity of multimodal imaging in detecting early, functional, and reversible alterations of the corneal epithelium and ocular surface. Importantly, imaging findings were used for descriptive and monitoring purposes and did not independently determine CTCAE grading, which was primarily driven by symptom severity and functional impairment, including BCVA reduction, in accordance with standardized toxicity reporting criteria.

Refractive analysis revealed transient changes in the majority of affected patients, predominantly characterized by new-onset or worsening irregular astigmatism, accompanied by minor spherical equivalent shifts. These refractive alterations correlated with temporary BCVA reduction and resolved in parallel with epithelial recovery, supporting their functional and reversible nature. Collectively, these observations suggest that MIRV-related visual impairment primarily reflects epithelial surface irregularity and tear film instability rather than permanent corneal damage.

Our findings are consistent with prior imaging studies describing ADC-related microcystic keratopathy as an off-target effect of maytansinoid payloads such as DM4, which preferentially affect rapidly dividing basal corneal epithelial cells [11,12,13]. Although the precise mechanisms remain incompletely elucidated, proposed pathways include nonspecific cellular uptake and disruption of microtubule dynamics, leading to impaired epithelial turnover. The early onset and reversibility of the observed changes further support a dose-dependent, non-structural epithelial disturbance. These observations also support the concept that corneal epithelial toxicity may represent a class effect of maytansinoid-containing antibody–drug conjugates.

The ophthalmologic management strategy adopted in this study—based on systematic lubrication, selective short courses of low-dose topical corticosteroids, and adjunctive surface-protective agents—proved effective in controlling ocular symptoms and instrumental alterations while allowing uninterrupted oncologic treatment. This approach aligns with emerging recommendations for the management of ADC-related ocular toxicity [10,12,13], which emphasize early detection, aggressive ocular surface protection, and judicious use of topical steroids.

Our experience underscores the critical role of ophthalmologists within the multidisciplinary care team for patients receiving MIRV. Structured baseline assessment, regular per-cycle evaluations, and patient education are essential to minimize functional impact and prevent unnecessary treatment interruptions. Furthermore, multimodal imaging—particularly AS-OCT and corneal topography—provides valuable complementary information beyond symptom-based grading alone, facilitating earlier recognition of subclinical changes.

Several limitations should be acknowledged. The study’s single-center design, relatively small sample size, and limited follow-up duration restrict generalizability and preclude evaluation of late or cumulative ocular effects. Additionally, the absence of a control group limits causal inference regarding the effectiveness of prophylactic strategies; however, withholding ocular surface protection in this vulnerable oncologic population would raise ethical concerns. Prior chemotherapy exposure may also have contributed to baseline ocular surface vulnerability and represents a potential confounding factor that could not be fully isolated in this observational design.

Future research should aim to better elucidate the mechanisms underlying MIRV-related ocular toxicity and to optimize preventive strategies. In particular, investigation of local drug exposure at the ocular surface may offer further insights. Exploratory ongoing work at our institution is assessing the presence of DM4 in tear fluid as part of future research perspectives, which may help clarify exposure-toxicity relationships and guide tailored prophylactic approaches [13]. Comparative studies evaluating standardized ophthalmologic management protocols are also warranted to further improve long-term ocular safety in patients treated with ADCs.

## 5. Conclusions

Corneal involvement represents the most distinctive ocular adverse event associated with mirvetuximab soravtansine. In this retrospective observational study, we documented a high frequency of microcystic epithelial keratopathy in patients with FRα-positive, platinum-resistant gynecologic malignancies undergoing MIRV therapy. Importantly, despite its prevalence, epithelial keratopathy proved to be transient, reversible, and clinically manageable when systematic ophthalmologic surveillance and preventive strategies were consistently applied.

The close correlation observed between patient-reported symptoms, multimodal imaging findings, and refractive changes highlights the value of an integrated diagnostic approach. Anterior segment OCT and corneal topography emerged as sensitive tools for early detection of epithelial irregularities and for distinguishing functional, reversible astigmatism from structural corneal alterations. Tear-film analysis further contributed to identifying surface instability as an early marker of ocular involvement. Together, these modalities support a dynamic model of assessment that complements standard CTCAE grading and allows for earlier recognition and more tailored management of ocular toxicity.

The therapeutic strategy adopted, combining intensive preservative-free lubrication, short courses of low-dose topical corticosteroids, and adjunctive antioxidant support, was effective in controlling ocular symptoms and instrumental alterations, while preserving oncologic treatment continuity. The absence of grade ≥ 3 ocular events or permanent vision loss in our cohort confirms that MIRV-related ocular toxicity can be safely managed within a structured ophthalmologic care pathway.

From a broader clinical perspective, our experience underscores the necessity of dedicated ophthalmologic monitoring pathways in modern oncology. Close collaboration between oncologists and ophthalmologists enables prompt detection of ocular changes, individualized management, and preservation of visual function, ultimately improving patient quality of life. This multidisciplinary model, integrating clinical expertise with advanced imaging technologies, should be considered an essential component of care for patients receiving antibody–drug conjugates.

Future research should focus on clarifying the pathophysiological mechanisms underlying ADC-related corneal toxicity and on optimizing preventive strategies. Exploratory investigations into local ocular surface exposure—such as the assessment of drug presence in tear fluid—represent promising future research directions and may contribute to refining prophylactic approaches. Advancing translational research in this field will not only improve the ocular safety profile of MIRV but also inform the development and clinical implementation of next-generation ADCs.

In conclusion, MIRV-associated ocular events represent a new but manageable challenge in contemporary ophthalmic practice in oncology. With vigilant monitoring, proactive surface protection, and close interdisciplinary collaboration, these complications can be effectively controlled, allowing patients to fully benefit from the therapeutic potential of targeted anticancer agents.

## Figures and Tables

**Table 1:** OAEs associated with MIRV: comparison between pivotal trials (SORAYA and MIRASOL) and the present cohort. The table highlights the higher detection rate of corneal epithelial toxicity observed with systematic ophthalmologic monitoring.

OAE	Pivot Trials (SORAYA/MIRASOL) All Grades (%)	Present Cohort (*n* = 31)
Blurred vision	~41%	28/31 (90.3%)
Microcystic epithelial keratopathy	29–36%	28/31 (90.3%)
Dry eye	25–28%	19/31 (61.3%)
Photophobia	~13%	20/31 (64.5%)
Any ≥ G3 ocular AE	6–9%	0/31 (0%)

**Table 2:** Changes in visual function, refractive parameters, and tear film stability during MIRV treatment and follow-up. BCVA, refractive parameters, and NIBUT are reported at baseline, at the worst point observed during treatment, and at follow-up. Values are expressed as median (Snellen) for BCVA, mean ± SD (range) for cylindrical refraction, and mean values for NIBUT.

Parameter	Baseline	During Treatment	Last Available Follow-Up
BCVA (Snellen, median)	20/20	20/32	20/25
Cylindrical refraction (D) mean ± SD (range)	−0.05 ± 0.15 (−0.50 to 0.00)	−1.61 ± 0.80 (−3.50 to −0.50)	−0.55 ± 0.42 (−1.50 to 0.00)
NIBUT (s, mean)	~10	~4	~7

## Data Availability

The data presented in this study are available on request from the corresponding author.
